# Radioimmunotherapy for CD133(+) colonic cancer stem cells inhibits tumor development in nude mice

**DOI:** 10.18632/oncotarget.16868

**Published:** 2017-04-06

**Authors:** Dinghu Weng, Xueyan Jin, Saimei Qin, Xiaoli Lan, Chong Chen, Xun Sun, Xianliang She, Changling Dong, Rui An

**Affiliations:** ^1^ Department of Nuclear Medicine, Union Hospital, Tongji Medical College, Huazhong University of Science and Technology, Hubei Province Key Laboratory of Molecular Imaging, Wuhan 430022, China; ^2^ Department of Neurosurgery, Renmin Hospital, Hubei University of Medicine, Shiyan 442000, China

**Keywords:** radioimmunotherapy, cancer stem cells, CD133, iodine-131, N-succinimidyl 3- (tri-n-butylstannyl) benzoate

## Abstract

Accumulating evidence indicates that cancer stem cells (CSCs) are the cause of tumor drug/radio-resistance or distant metastasis; therefore, it is essential to eliminate CSCs to cure cancer completely. The purpose of this study was to utilize radioimmunotherapy (RIT) to target CD133(+) colonic CSCs and observe whether this prevented tumor development, by assessing the maximum tolerated dose (MTD) of HCT116 tumor-bearing nude mice with escalating doses of ^131^I-AC133.1 monoclonal antibody (mAb), and determining the therapeutic efficacy of RIT with ^131^I-AC133.1 mAb. For RIT trials, animals were randomly divided into 4 groups of 6 per group, and injected with ^131^I-AC133.1 mAb (16.65 MBq/100 μl), AC133.1 mAb (173.1 μg/100 μl), saline (100 μl), or unrelated IgG1 as an isotype control. Iodine-131 was radiolabeled to AC133.1 mAb by conjugation with N-succinimidyl 3-(tri-n-butylstannyl) benzoate. The MTD of HCT116 tumor-bearing nude mice was 16.65 MBq. Both of the tumor volume doubling time and the survival time of the ^131^I-AC133.1 mAb group were significant longer than other groups (*P* < 0.001). CD133 expression was assessed by flow cytometry. Protein levels of cancer stem-like biomarkers (CD133, ALDH1, Lgr5, Vimentin, Snail1), and the proliferative rate of ^131^I-AC133.1 mAb group were lower than other groups (*P*<0.001); while its protein level of E-cadherin was higher than other groups. Furthermore, a large proportion of tumor necrosis was also observed in the ^131^I-AC133.1 mAb group, suggesting that RIT can destroy CSCs and effectively inhibit tumor development.

## INTRODUCTION

Colorectal cancer (CRC) is the second and third most common cancer worldwide in females and males, respectively [1]. It has the fifth highest cancer mortality rate in China and is the third leading cause of cancer death in the United States [2, 3]. The five-year survival rate of CRC was less than 70%, although neo-adjuvant chemotherapy has been improved [4], failure of anti-cancer therapy is attributed to a subpopulation of cells called cancer stem cells (CSCs) [5, 6].

CSCs play an important role in maintaining tumor growth through their unlimited self-renewal, therapeutic resistance, and the capacity to propagate tumors through asymmetric cell division [7]. Since CSCs were firstly isolated in acute myeloid leukemia [8], many CSCs of solid tumors, including breast, brain, lung, colon cancers as well as melanoma, have been identified [7]. Cell surface markers have been intensively used to isolate and identify CSCs, of which CD133 is frequently acknowledged as a CSC marker. CD133, also known as Prominin-1, is a 120-kDa cholesterol-interacting pentaspan-transmembrane glycoprotein that belongs to the Prominin family [9]. CD133 was first reported to be a CSC marker for brain tumors by Singh et al. [10, 11], following which O’Brien et al. [12] demonstrated that colonic CSCs within the CD133(+) population have stem cell potential, allowing self-maintenance and differentiation as well as reestablishing tumor heterogeneity upon serial transplantation.

Eradication of CSCs is critical for improvement in tumor prognosis [7, 13]. Various strategies have been proposed to target CSCs, including an antigen-based approach and inhibition of pathways related to self-renewal, maintenance and drug resistance of CSCs [14]. The antigen based approach relies on specific markers expressed on CSCs, with cognate antibody conjugated to drugs such as 5-Fluorouracil or radionuclide which could enhance the therapeutic effect of antibody-based immunotherapy alone. Jandal et al. [15] used ^188^Re-labeled anti-melanin monoclonal antibody (mAb) 6D2 for the treatment of mice bearing A2058 melanoma xenograft, indicating a similar rate of killing of melanoma stem cells and bulk cells. Then, Leyton et al. [16] utilized a radiolabeled antibody to target leukemia stem cells directly, demonstrating that high dose radioimmunotherapy (RIT) reduced leukemia stem cell viability and impaired self-renewal. Moreover, AI-Ejeh et al. [17] reported that ^177^Lu labeled anti-EGFR mAb alone significantly reduced the percentage of CD44(+)/CD24(-)/EpCAM(+) breast CSCs, especially when combined with chemotherapy and a poly ADP-ribose polymerase inhibitor. These findings suggest RIT has a potential advantage for targeting CSCs. To date, two products, Zevalin (^90^Y-ibritumomab tiuxetan) and Bexxar (^131^I-tositumomab) have been approved for the treatment of non-Hodgkin B-cell lymphoma in the clinic.

Beta (β)-emitters are commonly used for RIT based on their unique characteristics: radiolabeled antibodies conjugated to β-emitters can kill surrounding cells by a bystander “cross-fire” effect, which is independent of the expression of the target antigen or the sensitivity of the cells to chemotherapeutic drugs [18]. The physical properties of iodine-131 (^131^I) have allowed it to be widely used for RIT. However, dehalogenation is the main drawback of antibody radioiodination using the Iodogen method [19], which can decrease the stability of labeled antibody *in vivo* and cause radioactive damage to the thyroid. A potential factor contributing to the dehalogenation of proteins *in vivo* is the recognition of labeled iodophenyl groups on the protein by deiodiases known to be involved in the metabolism of thyroid hormones [20]. Santous et al. [21] verified that retention labeling such as N-succinimidyl 3-(tri-n-butylstannyl) benzoate (ATE) method have a fast blood clearance, better target organ/background relation and low uptake in the thyroid and stomach compared with the direct labeling method. Our previous work utilized ^131^I labeled AC133.1 mAb to trace CD133(+) colonic CSCs *in vivo* [22], which established a foundation for RIT of CSCs. In this study, we attempted to target CD133(+) colonic CSCs with RIT.

## RESULTS

### MAb characterization

The retention time of N-succinimidyl 3-[131I]iodobenzoate (^131^I-SIB) was approximately 11.0 min (data not shown), and generated about 88.0% yield and 60.4 to 76.6% of this intermediate was coupled to AC133.1 mAb following a 30 min reaction at room temperature (RT). The radiospecific activity of ^131^I-AC133.1 mAb was 77.70 KBq/μg to 96.20 KBq/μg, Scatchard analyses of the binding data revealed that the equilibrium dissociation constant K_D_ for AC133.1 mAb was (4.76 ± 0.30) × 10^-8^ M, and the nonspecific binding was less than 2%.

**Figure 1:**
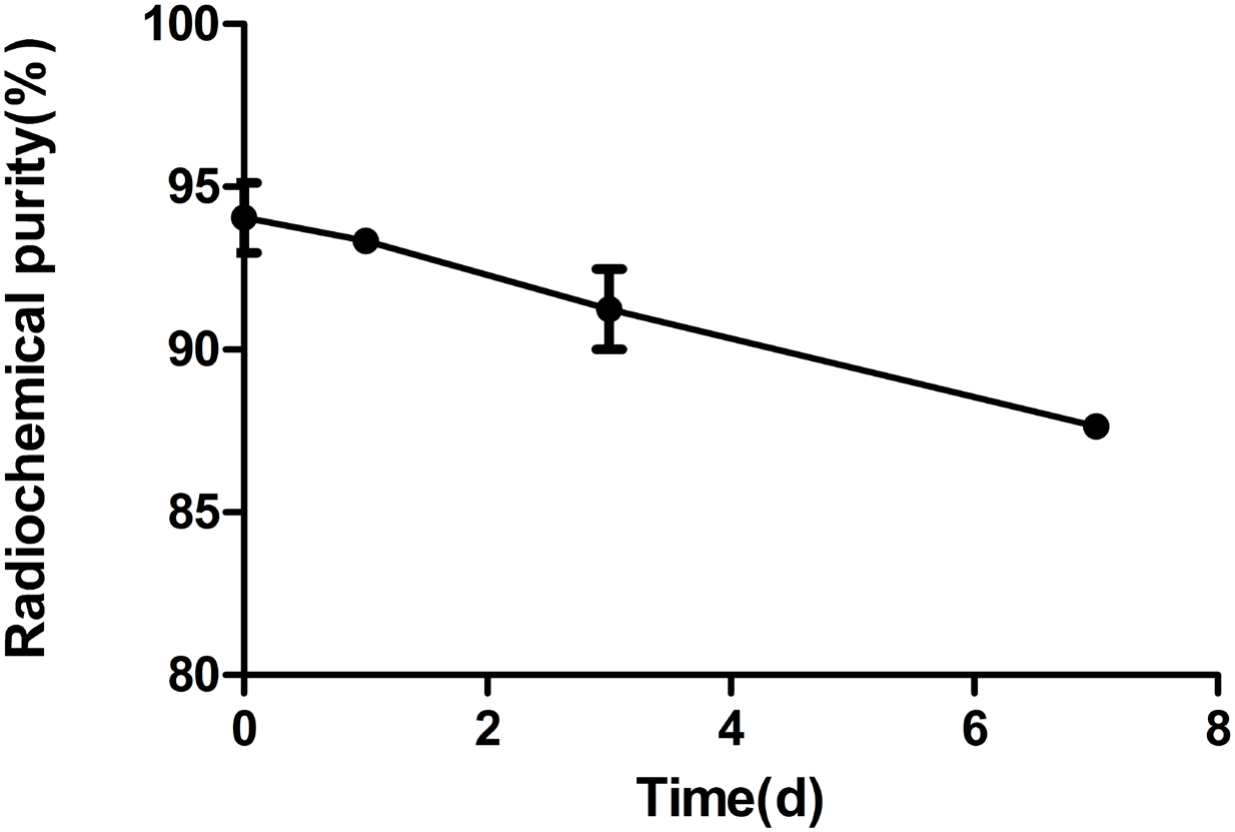
The stability of ^131^I-AC133.1 mAb in FBS The radiochemistry purity of ^131^I-AC133.1 mAb at different time points (0, 1, 3, and 7d).

The radiochemical purity of the labeled antibody was 94.05 ± 1.53% and it was 87.64 ± 0.33% at day 7 in fetal bovine serum (FBS) (Figure 1).

### Does escalation and toxicity evaluation

For the doses 7.40, 9.25, 11.10, 12.95 and 14.80 MBq, there were no related deaths or loss of weight exceeding 20% of its initial. At the 16.65 MBq dose, two mice showed temporary weight loss in the first two weeks, but this did not exceed 20%. There was one case of discomfort and weight loss exceeding 20% at day 18 at a dosage of 18.50 MBq. However, for the doses of 20.35 and 22.20 MBq, animals were showed weight loss greater than 20% or death within 20 d. Based on these results, we determined the maximum tolerated dose (MTD) of HCT116 bearing-nude mice was 16.65 MBq.

### Therapeutic response

**Figure 2:**
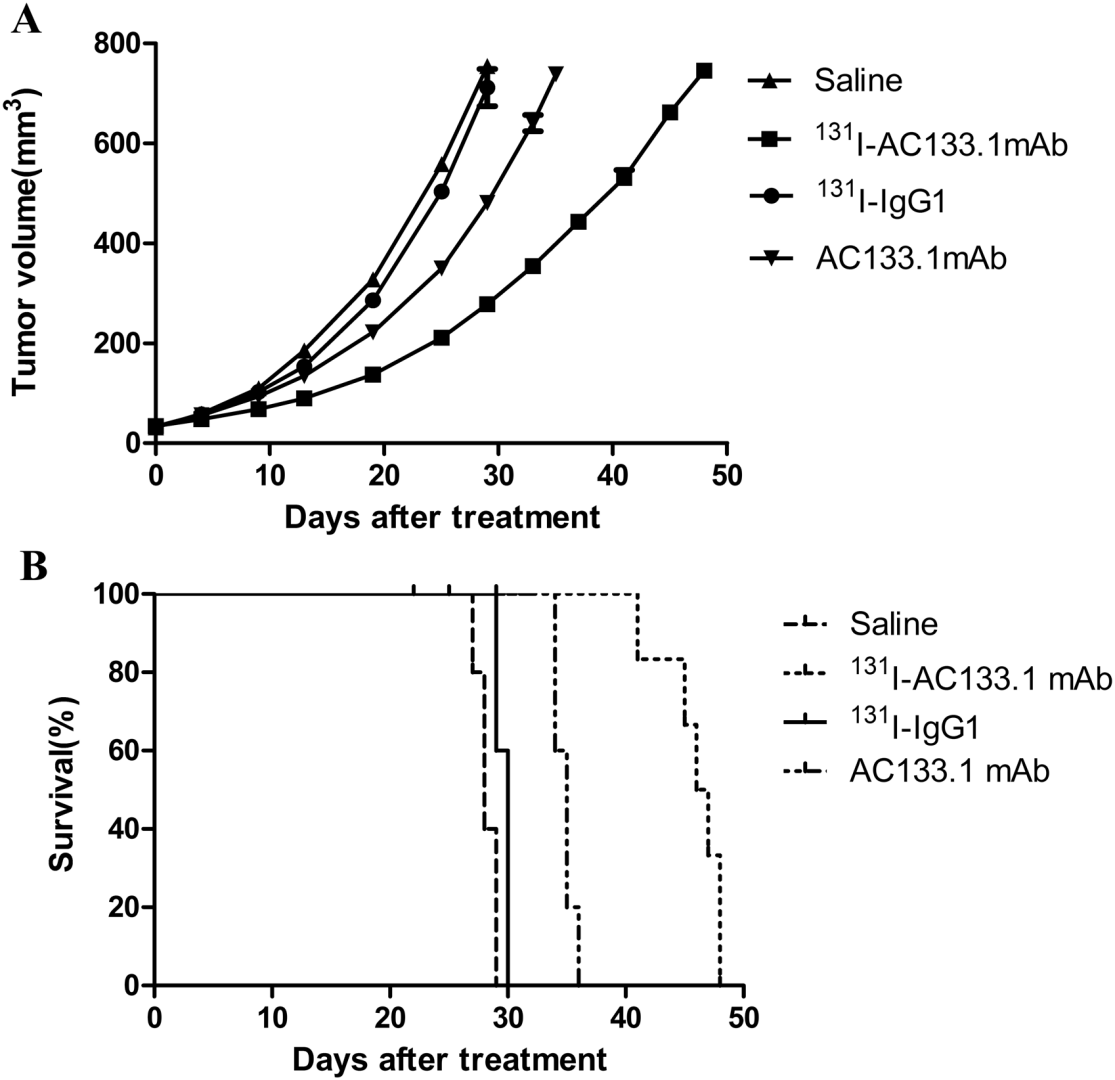
Tumor growth curves **(A)** and survival curves **(B)** of the four groups. Initial tumor sizes at the time of treatment ranged from 33.07 ± 4.94 mm^3^ (diameter ~4 mm), both the tumor VDT (A) and the survival time (B) of ^131^I-AC133.1 mAb group were longer than the controls (*P* < 0.001); there were no significant differences for VDT (P=0.071) and survival time (P=0.420) among the controls.

Initial tumor sizes at the time of treatment ranged from 33.07 ± 4.94 mm^3^ (diameter ~4 mm), but the difference in tumor volume and weight of tumor-bearing nude mice were not statistically significant between the groups. From the results of tumor growth curve (Figure 2A), we conducted the tumor volume doubling time (VDT) of the four groups. A statistically significant difference was observed for tumor VDT between the ^131^I-AC133.1 mAb group and other three groups (*P*<0.001), with a value of 9.36 ± 0.45 days for the ^131^I-AC133.1 mAb group, and 6.29 ±0.78 days, 6.42 ± 0.35 days and 6.89 ± 0.30 days for saline, ^131^I-IgG1 and AC133.1 mAb groups, respectively. There were no significant differences among the other groups (P=0.071). From the survival curve (Figure 2B), we calculated the median survival time of each group, and they were 44.0 d, 27.8 d, 29.4 d, 32.6 d for ^131^I-AC133.1 mAb, Saline, ^131^I-IgG1, AC133.1 mAb groups, respectively; and the survival time of ^131^I-AC133.1 mAb group was longer than the other groups (*P* < 0.001), there were no significant differences among the controls (P=0.420).

### Biodistribution measurement and radiation dosimetry

The highest ratio of tumor/thyroid was 19.12 ± 3.21 at day 7, which was higher compared with our previous study using the Iodogen method. Nontarget organs such as muscle, bone, gastrointestinal, lungs, and heart all maintained low levels of ^131^I-AC133.1 mAb. From the biodistribution data (Table 1), we calculated the accumulated radiation dosimetry of tumor in ^131^I-AC133.1mAb group to be 5,966.34 ± 54.90 cGy.

**Table 1:** The biodistribution of ^131^I-AC133.1 mAb in HCT116 tumor-bearing nude mice (Data were shown as mean ± SD, %ID/g, n=4)

Tissue	1 day	3 day	5 day	7 day	9 day
Tumor	4.63 ± 0.07	8.69 ± 0.34	7.28 ± 0.11	6.52 ± 0.16	3.15 ± 0.16
Blood	16.87 ± 0.60	10.72 ± 1.80	8.20 ± 0.21	7.48 ± 0.66	4.77 ± 0.58
Femoral bone	1.88 ± 0.31	1.31 ± 0.36	1.15 ± 0.19	0.76 ± 0.03	0.70 ± 0.03
Muscle	1.23 ± 0.12	0.97 ± 0.29	0.81 ± 0.17	0.60 ± 0.09	0.35 ± 0.04
Liver	8.62 ± 0.48	9.51 ± 0.72	7.91 ± 0.71	5.35 ± 0.82	2.58 ± 0.37
Kidney	2.58 ± 0.37	9.97 ± 0.76	5.74 ± 0.74	4.07 ± 0.87	1.99 ± 0.10
Intestine	1.16 ± 0.08	1.27 ± 0.19	0.91 ± 0.12	0.67 ± 0.11	0.41 ± 0.10
Spleen	6.51 ± 0.34	6.63 ± 0.44	2.83 ± 0.16	2.44 ± 0.06	1.21 ± 0.06
Stomach	1.14 ± 0.02	1.12 ± 0.11	0.99 ± 0.04	0.66 ± 0.04	0.43 ± 0.08
Lung	8.48 ± 1.16	5.31 ± 0.86	4.51 ± 0.15	3.94 ± 0.45	2.43 ± 0.30
Heart	4.70 ± 0.04	2.73 ± 0.60	1.83 ± 0.09	1.28 ± 0.18	0.82 ± 0.05
Thyroid	0.99 ± 0.12	0.82 ± 0.05	0.74 ± 0.01	0.35 ± 0.05	0.34 ± 0.03
Brain	0.60 ± 0.10	0.37 ± 0.12	0.30 ± 0.06	0.22 ± 0.06	0.17 ± 0.03
Tumor/muscle	3.78 ± 0.30	9.51 ± 2.46	9.24 ± 1.73	7.80 ± 1.42	6.63 ± 1.14
Tumor/thyroid	4.69 ± 0.50	10.65 ± 0.74	9.79 ± 0.08	19.12 ± 3.21	9.26 ± 0.29
Tumor/blood	0.28 ± 0.03	0.82 ± 0.12	0.89 ± 0.04	0.88 ± 0.09	0.66 ± 0.04

### Flow cytometry, western blot (WB), hematoxylin and eosin (H&E) and Ki67 staining

**Figure 3:**
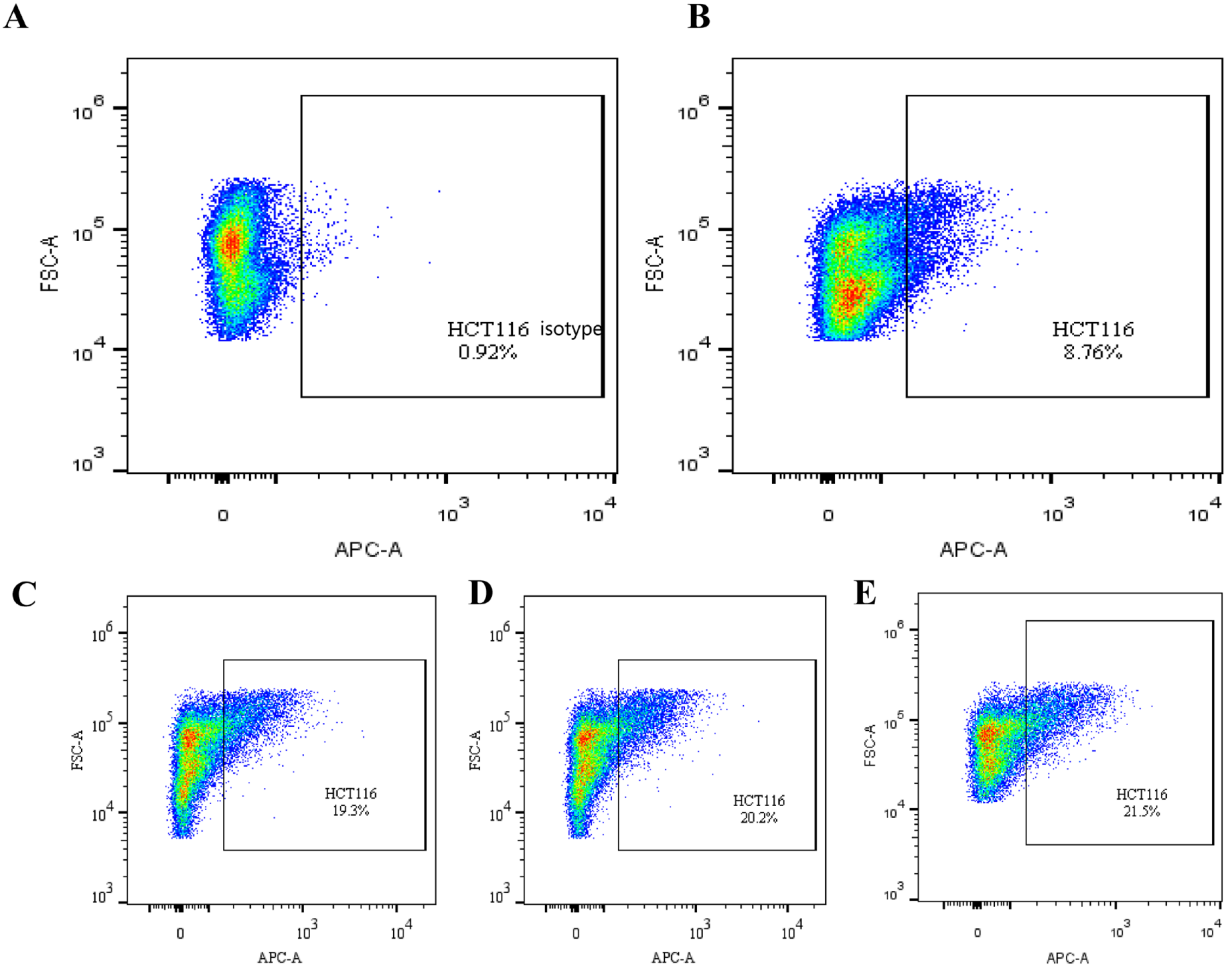
The CD133 expression tested by flow cytometry of the four groups **(A)** Isotype control of flow cytometry. **(B)** ^131^I-AC133.1 mAb group, 7.15 ± 0.95%; **(C)** ^131^I-IgG1group, 20.12 ± 1.46%; **(D)** AC133.1 mAb group, 18.24 ± 2.53%; **(E)** Saline group, 21.70 ± 1.61%. The CD133 expression of ^131^I-AC133.1 mAb group was lower than the controls (*P* < 0.001), and there were no significant differences among the other three groups (P=0.244).

**Figure 4:**
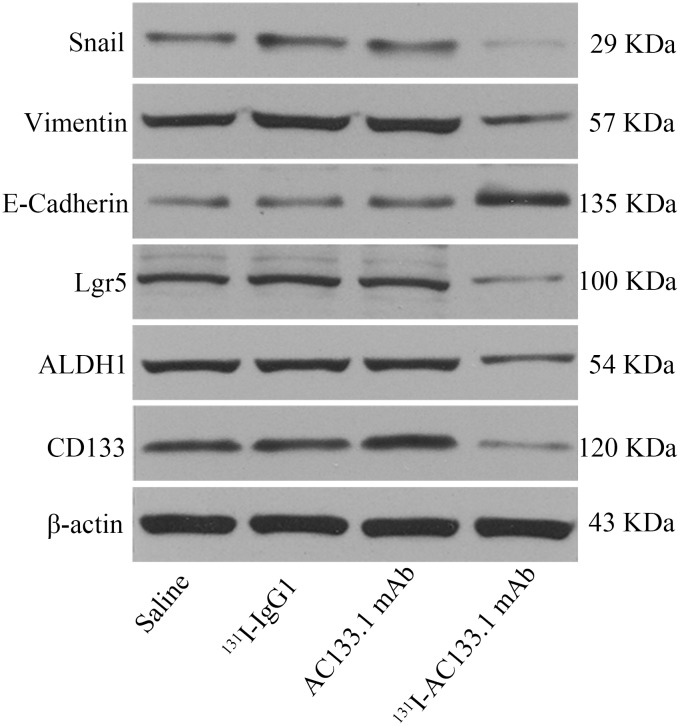
The protein level of cancer stem-like markers Cancer stem-like biomarkers include CD133, ALDH1, Lgr5, E-cadherin, Vimentin, Snail1. The expression of CD133, ALDH1, Lgr5, Vimentin and Snail1 in ^131^I-AC133.1 mAb group were lower than other groups, while the level of E-cadherin was higher.

**Figure 5:**
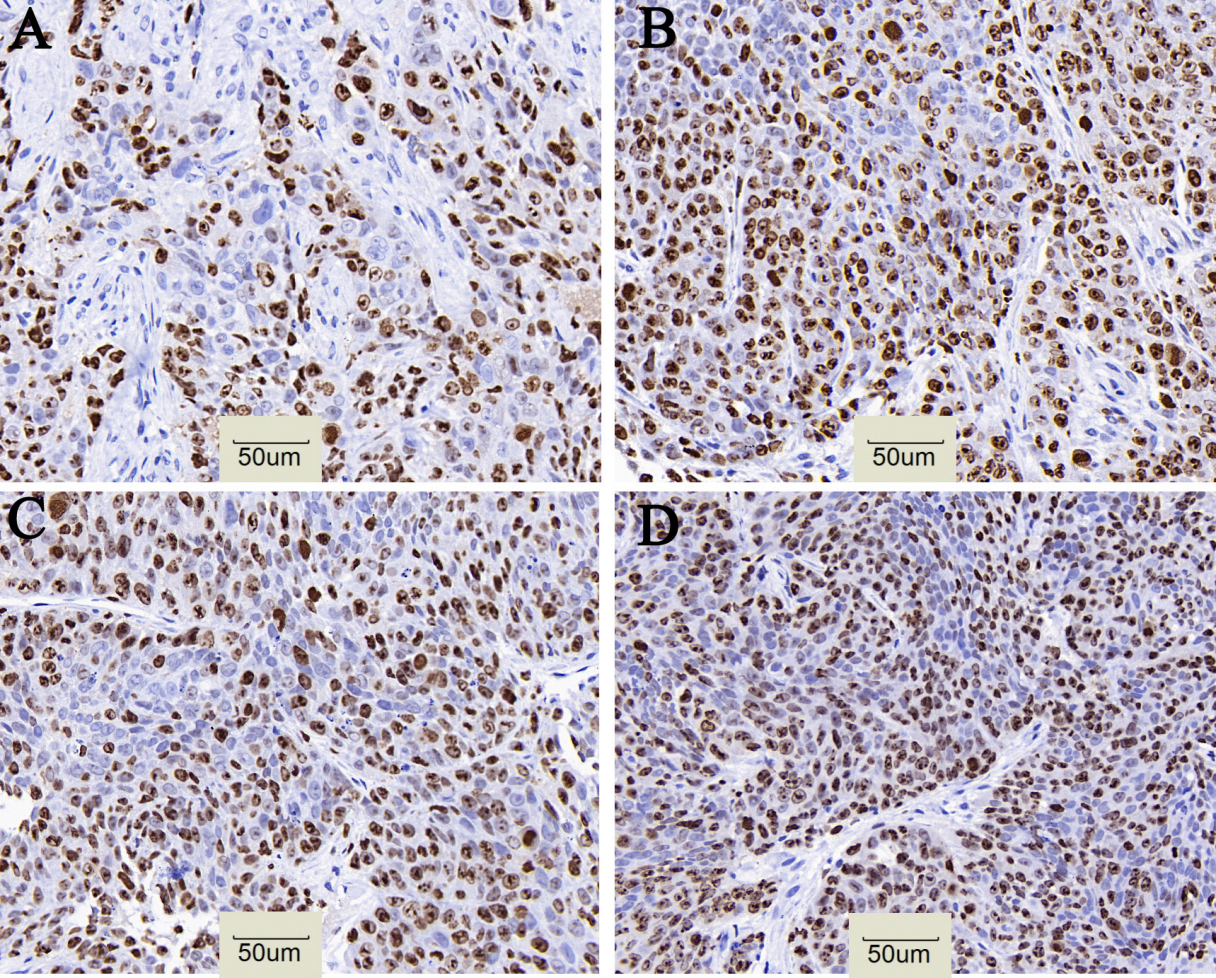
Cell proliferative index of tumor in four groups **(A)** ^131^I-AC133.1 mAb group, 9.52 ± 1.33%; **(B)** ^131^I-IgG1 group, 37.46 ± 3.72%; **(C)** AC133.1 mAb group, 32.60 ± 2.88%; **(D)** Saline group, 45.56 ± 3.52%. The proliferative index of ^131^I-AC133.1 mAb group was lower than the controls (*P* < 0.001), (×200).

**Figure 6:**
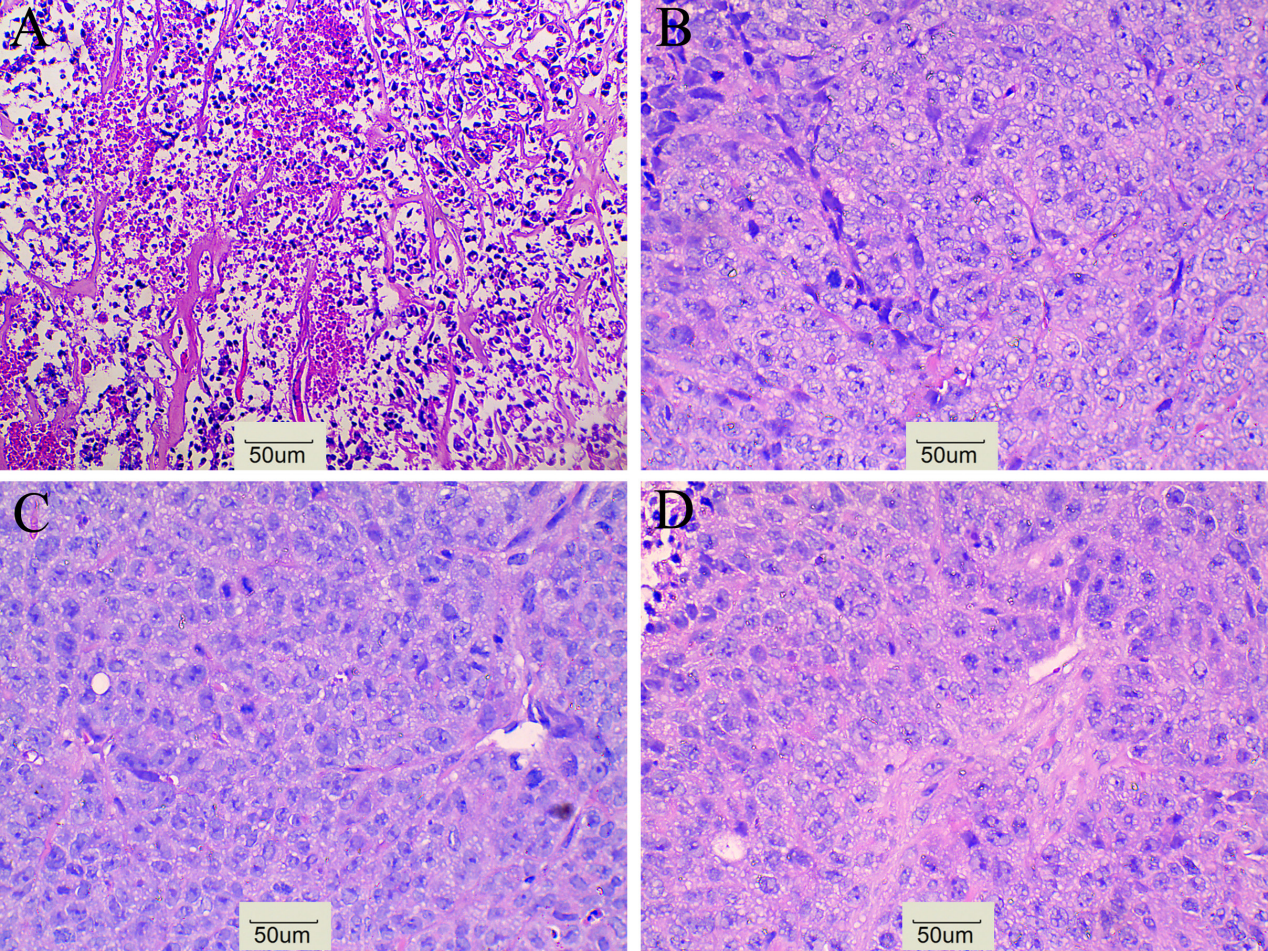
H&E staining **(A)** ^131^I-AC133.1 mAb group, **(B)** ^131^I-IgG1 group, **(C)** AC133.1 mAb group, **(D)** Saline group. H&E staining shows a large necrosis in ^131^I-AC133.1 mAb group, while no obvious necrosis were observed in control groups (×200).

CD133 expression in the ^131^I-AC133.1mAb group was 7.15 ± 0.95%, which was significant lower than the controls (saline group: 21.70 ± 1.61%, ^131^I-IgG1 group: 20.12 ± 1.46%, AC133.1 mAb group: 18.24 ± 2.53%) (*P* < 0.001) (Figure 3), and there were no significant differences among the controls (P=0.244). Protein levels cancer stem-like biomarkers (CD133, ALDH1, Lgr5, E-cadherin, Vimentin, Snail1) were also tested, indicating that the expression of CD133, ALDH1, Lgr5, Vimentin and Snail1 in the ^131^I-AC133.1 mAb group were lower than the other groups, while the level of E-cadherin was higher (Figure 4). Ki67 analysis showed reduced proliferative index in the ^131^I-AC133.1 mAb group (9.52 ± 1.33%), whereas the ^131^I-IgG1, AC133.1 mAb, saline groups had significantly higher proliferative indices (37.46 ± 3.72%, 32.60 ± 2.88%, 45.56 ± 3.52%, respectively) (*P*<0.001) (Figure 5). H&E staining of tumor tissues showed a large necrotic area in tumors from the ^131^I-AC133.1 mAb group; however, no obvious necrosis was observed in the controls (Figure 6).

## DISCUSSION

In this study, AC133.1 mAb was radioiodinated by the ATE method and showed satisfactory characteristics. The ^131^I-AC133.1 mAb was utilized to target CD133(+) colorectal CSCs, and analysis of the therapeutic effect (tumor VDT, median survival time, CD133 expression by flow cytometry, protein levels of cancer stem-like biomarkers, Ki67 and H&E staining of tumors) suggested that ^131^I-AC133.1 mAb could destroy CSCs and inhibit tumor development.

There is an urgent need to eliminate CSCs based on their critical role in tumor relapse and distant metastasis after therapy; however, conventional therapeutics have no effect on CSCs due to their chemo/radio-resistance. Therefore, some studies have attempted to target CSCs with RIT. Jandl et al. [15] utilized an ^188^Re-6D2 antibody to target mice bearing A2058 melanoma xenografts, showing RIT killed the melanoma stem cells at the same rate as the other tumor cells. Later, Leyton et al. [23] applied ^111^In labeled anti-CD123 mAb, CSL360, modified with nuclear translocation sequence peptides to study the acute myeloid leukemia engraftment assay in the prevalent NOD/SCID mouse, demonstrating a significant decrease in CD123(+) leukemic stem cells and impaired self-renewal was achieved with high doses of RIT. Both experiments suggested that RIT may be a useful therapeutic modality for eradicating CSCs. To target CSCs, a good CSCs marker is required.

To date, many cell surface markers including CD133, CD44, EpCAM, CD24, CD29, Lgr5, CD166, Sox2, and Oct4, either separately or in combination, have been used to identify colorectal CSCs [9, 24–26]; however, CD133 is the most frequently used. O’Brien et al. [12] and Lucia et al. [27] foundthatcolonic CSCs were enriched in CD133(+) cells and that their tumorigenic ability was higher than that of CD133(-) cells, indicating CD133 could be used as a useful biomarker for colonic CSCs. Our previous study utilized ^131^I-AC133.1 mAb to trace CD133(+) colonic CSCs successfully *in vivo* [22], which laid a solid foundation for the use of RIT to target CD133(+) colonic CSCs with radioiodinated compounds.

For radioiodination mAb, it is generally assumed that the loss of radioiodine from these proteins following their *in vivo* administration can be attributed to the action of dehalogenases normally involved in the metabolism of thyroid hormones [20], thus affecting the stability of the radiolabeled antibody *in vivo*. However, using retention labeling such as ATE method can avoid dehalogenation [19, 28]. ATE contains an active ester that can be combined with ^131^I quickly; the labeling rate of ^131^I-SIB was about 88.5%, which allows the intermediate to conjugate with AC133.1 mAb under appropriate conditions. The radiochemistry purity of ^131^I-AC133.1 mAb was 94.05 ± 1.53%, and was 87.64 ± 0.33% in FBS at day 7, indicating the radiolabeled compound was relatively stable. In a cell saturated assay, the equilibrium dissociation K_D_ was (4.76 ± 0.30) × 10^-8^ M, revealing the radiolabeled antibody had a good affinity for its antigen. The radiospecific activity of labeled antibody was 77.70 KBq/μg to 96.20 KBq/μg, which was lower than our previous report using Iodogen method [22], as the ATE method involved a two-step reaction. Its enhanced characteristics *in vitro* indicated the feasible use of RIT *in vivo* with ^131^I-AC133.1 mAb.

To choose an appropriate dosage, we performed MTD assay by assessing the health status and weight of mice. The initial dose of MTD experiment was 7.40 MBq escalating in 1.85 MBq intervals, to a final dose of 22.20 MBq. None of the mice loss of weight exceeds 20% of its initial or died within 30 days, up to and including a dose of 16.65 MBq. However, at least one of these events emerged over a dose exceeding 16.65 MBq. We thus determined the MTD to be 16.65 MBq. For RIT experiments, the initial tumor volume was 33.06 ± 4.98 mm^3^ with a diameter of ~4 mm. This diameter was chosen because the tissue penetration of ^131^I is less than 3 mm [29, 30], and our preliminary experiments demonstrated that the radiolabeled mAb had no obvious effect on tumor development if its initial volume was too large. Generally, RIT plays a role mainly dependent on the following effects: antibody dependent cellular cytotoxicity (ADCC), antigen-antibody complexes activating the complement pathway, and the killing effect of radionuclide for the targets. Radiotherapy causes DNA damage directly by ionization or indirectly via the generation of reactive oxygen species (ROS), therapy destroying the targets. As ^131^I-AC133.1 mAb could destroy CD133(+) CSCs specifically, the CD133 expression was confirmed by flow cytometry and the protein level of CD133 in ^131^I-AC133.1 mAb group were found to be lower than in the control groups. The percentage of CSCs was reduced, resulting in slowed tumor growth, reduced proliferative index, and longer VDT and survival time, thus supporting our hypothesis. Moreover, the protein level of other stem-like markers were tested, the expression of Lgr5, ALDH1, Vimentin and Snail1 in ^131^I-AC133.1 mAb group were lower than other observed in the other groups, while the level of E-cadherin was higher, as the expression of E-cadherin is negatively correlated with tumor invasion and metastasis. There was no obvious therapeutic effect in the AC133.1 mAb group; one of the reasons for this may be that in the BALB/c nude mouse model, the effect of ADCC alone was so weak that it could not inhibit tumor development.

To calculate the radiation dosimetry, we performed biodistribution experiments. The highest ratio of tumor/thyroid tissue was 19.12 ± 3.27 at day 7, which was higher than our previous report using the Iodogen method [22], verifying the ATE method has a better stability *in vivo*. A sufficient radiation dose delivered to the tumor is important for the curative effect, and tumor responses will depend on the amount of tissue being irradiated as well as the radiation sensitivity of the tissue, because solid tumors typically require 3,500-10,000 cGy for a meaningful response [31], consistent with our result (5,966.34 ± 54.90 cGy).

A number of biomarkers of colorectal CSCs have been identified, and it is thought that only a small subset of CD133(+) cells were colorectal CSCs. CD133 is a commonly used colorectal CSC marker, and can be used in combination with other markers to screen the purity of CSCs. Haraguchi et al. [32] identified cancer initiating cells were more likely to be from a CD133(+)CD44(+) population compared with CD133(+) or CD44(+) cells alone. For RIT, the therapeutic effect was highly relevant to the radionuclide. ^131^I has a low linear energy transfer (LET = 0.1-1.0 kev/μm), thus producing non-specific exposure to normal organs as a result of the cross-fire effect. Alpha particles have high linear energy transfer, and lethal effect does not rely completely on tissue oxygen; thus is has been commonly used for RIT in recent years [33–35]. Furthermore, the poor pharmacokinetic properties caused by the high molecular weight of mAbs (~150kDa) limits the effects of RIT; however, progress in genetic engineering antibody, and strategies including pretargeted technology [29], fractionated RIT [31, 36], and RIT combination with other therapeutic modalities such as chemotherapy, mAb based biotherapy, and customized radiolabeled antibody cocktails might enhance the effect of RIT [37–42]. In conclusion, our results indicate that mAb-conjugated RIT successfully retards tumor progression, while enhancing median survival time by targeting CD133(+) CSCs in a mouse model of colon cancer.

## MATERIALS AND METHODS

### Ethics statement

All animal procedures were performed according to a protocol approved by the Institutional Animal Care and Use Committee of HuaZhong University of Science and Technology.

### Mab and cells

AC133.1 mAb preparations were produced from ascites grown from BALB/c mice that received intraperitoneal injections of the appropriate hybridoma (HB-12346). AC133.1 mAb is an IgG1 murine monoclonal antibody purified by protein A/G. HCT116 cells were purchased from the Cell Bank of the Chinese Academy of Sciences, grown in RPMI1640 and cultured in medium supplemented with 10% (v/v) FBS at 37°C in a humidified atmosphere with 5% CO_2_.

### Antibody labeling using the ATE method

ATE was synthesized according to a previous report [43]. For this procedure, 0.9 μl ATE was placed into a reaction vial fitted with a septum containing 100 μl 1% HOAC/MeOH, 10 μl N-chlorosuccinimide in MeOH, and 10 μl of phosphate buffered saline (PBS). The desired quantity of Na^131^I was added (about 38.48 MBq), and after 10 min at RT the reaction was quenched by the addition of 10 μl of 0.72 mg/ml aqueous solution of NaHSO_3_ solution. The intermediate product, ^131^I-SIB, was purified by sepax positive-phase semipreparative column (HPLC), and ^131^I-SIB was dried by N_2_. Then, 50 μl of AC133.1 mAb was dissolved in 50 μl borate buffer (pH = 8.7) and then added into the above dried ^131^I-SIB at RT for 30 min with constant shaking. The reaction was terminated by the addition of 300 μl of 0.2 M glycine. The crude radiolabeled compound was purified by a sephadex G-25 column using PBS as the eluent.

### Equilibrium dissociation constant determination

The equilibrium dissociation constant for the labeled AC133.1 mAb preparations to HCT116 were determined using a cell-saturated assay. Briefly, 1 × 10^6^ cells/well were grown to confluence in 12-well plates, serial dilutions of each labeled AC133.1 mAb preparations were added to each well in triplicate after 150 μl 1% BSA/PBS was added in advance and incubated for 30 min, and then co-incubated at 37°C for 4 h. The wells were washed 3 times with 1% BSA/PBS and 0.1 M NaOH solution was used to crack cells. The supernatant was collected, precipitated separately and radioactivity measured using an automated γ counter. Non-specific cell binding was determined by adding 500-fold unlabeled AC133.1 mAb, incubated for 30 min and then adding the above labeled AC133.1 mAb. The percentage of specific binding was calculated by subtracting the nonspecific binding from total binding. Equilibrium dissociation constants were analyzed using a computer program.

### Serum stability

^131^I-AC133.1 mAb was mixed with an appropriate volume FBS at 4°C, and the radiochemical purity was calculated at different time points (0, 1, 3, and 7 d).

### Tumor model

Male athymic mice (nu/nu genotype, BALB/c background) of at least 8 weeks of age and weighing 20–24 g were purchased from the Beijing HuaFuKang Company (Beijing, China). The HCT116 colonic tumor model was established by the injection of 2 × 10^6^ cells in 100 μl PBS into the lower right limbs. Tumor volumes were calculated as

(short dimension)^2^ × long dimension/2

Animals were randomized by tumor volume, and therapeutic trials were initiated when the tumor diameter was approximately 4 mm.

### RIT experiments

Two therapy studies were performed using ^131^I-AC133.1 mAb. The first therapy study was designed to assess the MTD in HCT116-bearing nude mice. The MTD, corresponding to the highest dose at which weight loss did not exceed 20% of initial weight, was determined by monitoring the weight and health status of tumor-bearing nude mice injected intravenously with increasing doses of ^131^I-AC133.1 mAb. Nine groups were treated with 7.40, 9.25, 11.10, 12.95, 14.80, 16.65, 18.50, 20.35 or 22.20 ^131^I-AC133.1 mAb, respectively (each dose was tested in six mice). Because organ damage may not develop immediately, animals were monitored for an addition 30 days. The second experiment studied the therapeutic efficacy of RIT (six mice per group). To ensure therapeutic efficacy, the ^131^I-AC133.1 mAb group received the MTD in a volume of 100 μl. For the AC133.1 mAb group, the amount of antibody was calculated from the radiospecific activity of the radiolabeled compound. Unrelated IgG1 was used as the isotype control. Animals were housed individually, and bedding was changed at 48 h and 96 h and twice weekly to reduce nonspecific background exposure. Tumor sizes were measured every 4–5 days and health status was observed daily. Mice in the RIT trials were excluded when weight loss exceeded 20% of starting weight, or the tumor volume was greater than 750 mm^3^ [44].

An antitumor effect was expressed as tumor VDT, which was calculated using the following equation: VDT = (T−T_0_) × log2/log (V/V_0_), where T−T_0_ indicates the length of time between two measurements and V_0_ and V denote the tumor volume at two points of measurements [45]. In addition, survival curves were constructed.

### Biodistribution and radiation dosimetry

Tissue distribution studies were initiated when the mean tumor diameter reached 0.8-1.0 cm and mice were then injected intravenously with about 7.40 MBq ^131^I-AC133.1 mAb. On days 1, 3, 5, 7, and 9 after injection, groups of 4 animals were euthanized, and tissues (including blood, tumor, muscle, femoral bone, spleen, kidney, liver, stomach, large intestine, lung, heart, thyroid, brain) were obtained and weighed. Results are expressed as a percentage of the injected dose. The radiation absorbed dose was calculated from the biodistribution data, and the percentage injected dose per gram (% ID/g) value was corrected for the radiation decay of ^131^I and converted to μci/g. The cumulative activity in tumors (μci-h/g) was calculated from the area under the resultant μci/g curves using the trapezoid integration method. A uniform distribution of ^131^I in tissues was assumed. The radiation absorbed dose in rads (cGy) received by tumor was calculated by multiplying its cumulative activity by the equilibrium absorbed dose constant (0.4313 g-rad/μci-h) of ^131^I [28]. Because of the low absorbed fractions of ^131^I γ-rays in small tissues such as those in BALB/c athymic mice, the dose due to γ exposure was not included.

### Flow cytometry, western blot, H&E, and ki67 staining

For flow cytometry, marginal tumor tissues not including necrotic tissues or blood streaks were sheared, washed three times with saline, cut into slices, digested with collagenase IV for 1 h at 37°C, filtered through a 70-μm screen mesh, then resuspended as a monolayer suspension. Then, 3 μl APC-CD133/1(MiltenyiBiotec) was added and incubated at 4°C for 30 min, centrifuged for 5 min at 110 g, and then resuspended in 300 μl PBS for sample testing.

After sacrificing the mice, the tumor tissues were removed immediately and ground completely. After centrifugation (400 × g, 4°C) for 5 min, the supernatant was collected to quantify the protein using the Bradford protein assay kit (Thermo Fisher). Protein was separated by sodium dodecyl sulfate-polyacrylamide gel electrophoresis and then electroblotted onto a polyvinylidenedifluoride membrane (Millipore, Billerica, MA, USA). The membrane was incubated with mAb (Miltenyi Biotec, Bergisch Gladbach, Germany) overnight at 4°C. β-actin was used as a control. Each experiment was performed in triplicate.

For H&E staining, tumor tissues were fixed in 4% paraformaldehyde for 10–12 h, embedded in paraffin, passed through a serial process of dewaxing, dyeing, hydration and drying.

For Ki67 staining, an antigen retrieval kit from MiltenyiBiotec was used according to the manufacturer's specifications. Endogenous peroxidase was blocked with 3% hydrogen peroxide in PBS and incubated with aprotein-blocking solution containing PBS (pH 7.5). Samples were incubated with a 1:200 dilution of Ki67, incubated in diaminobenzidine, mounted on glass slides with cover slips.

### Statistical analysis

Quantitative data were expressed as mean ± standard deviation (SD) or percentage when appropriate. All data were analyzed with the One-way ANOVA or nonparametric tests (Mann–Whitney *U*-test for comparison of 2 groups and the Kruskal–Wallis test for comparison of multiple groups). Survival was calculated using Kaplan-Meier curves. *P <* 0.05 was considered statistically significant.

## Abbreviations

CSCs: cancer stem cells
RIT: radioimmunotherapy
mAb: monoclonal antibody
MTD: maximum tolerated dose
CRC: colorectal cancer
WB: western blot
H&E: hematoxylin eosin
ADCC: antibody dependent cellular cytotoxicity
ROS: reactive oxygen species
LET: linger energy transfer
FBS: fetal bovine serum
RT: room temperature
HPLC: high performance liquid chromatography
PBS: phosphate buffered saline
SD: standard deviation.
